# Short-Term Symptom and Functional Outcomes Following Definitive Chemoradiotherapy Versus Surgery Plus Adjuvant Radiotherapy in Locally Advanced Oral Cavity Squamous Cell Carcinoma

**DOI:** 10.7759/cureus.111851

**Published:** 2026-06-30

**Authors:** Sabrina Taher, Anowarul Islam, Altaf Hossain, Mohammed Mehbub Ahsan Rony, Mohammad Omar Faruque, Cynthia Afreen, Anamika Das, Tasneem Hossain, Mehnaz Riffat Mou, Mohammad Mehedi Hasan Chowdhury

**Affiliations:** 1 Radiation Oncology, Evercare Hospital Dhaka, Dhaka, BGD; 2 Cardiology, National Institute of Cardiovascular Diseases, Dhaka, BGD; 3 Radiation Oncology, National Institute of Cancer Research and Hospital, Dhaka, BGD; 4 Radiation Oncology, Sylhet Muhammad Ataul Gani Osmani Medical College Hospital, Sylhet, BGD; 5 Radiation Oncology, Enam Medical College and Hospital, Dhaka, BGD; 6 Radiation Oncology, Rangpur Medical College Hospital, Rangpur, BGD; 7 Radiology, Bangladesh Medical University, Dhaka, BGD; 8 Surgical Oncology, National Institute of Cancer Research and Hospital, Dhaka, BGD

**Keywords:** adjuvant radiotherapy, chemoradiotherapy, fact-hnsi-10, functional outcomes, oral cavity cancer, quality of life, squamous cell carcinoma, symptom burden

## Abstract

Background

Patients with locally advanced oral cavity squamous cell carcinoma (OCSCC) often experience symptoms that interfere with eating, swallowing, comfort, and daily functioning. Surgery followed by adjuvant radiotherapy (RT) remains the standard treatment for operable disease, whereas definitive concurrent chemoradiotherapy (CCRT) is used selectively in patients who are unsuitable for, unwilling to undergo, or unable to undergo timely surgery.

Objective

This study compared short-term symptom burden and functional outcomes between patients with locally advanced OCSCC treated with definitive CCRT and those treated with surgery followed by adjuvant RT.

Methods

This prospective, non-randomized comparative cohort study was conducted at two cancer centers in Bangladesh from January 2023 to October 2024. Sixty-eight previously untreated patients with stage III or IVA OCSCC were included. Thirty-four patients received definitive CCRT, and 34 underwent surgery followed by adjuvant RT. Symptom burden and functional status were assessed using the Functional Assessment of Cancer Therapy Head and Neck Symptom Index-10-item version (FACT-HNSI-10). The primary outcome was the change in the FACT-HNSI-10-derived raw symptom burden score from baseline to post-treatment assessment. Secondary outcomes included changes in pain, dysphagia, bleeding or ulceration, and respiratory distress.

Results

FACT-HNSI-10-derived raw symptom burden scores decreased significantly after treatment in both groups, indicating improvement in symptom burden according to the scoring approach used in this study. The mean score decreased from 30.2±0.8 to 24.8±1.3 in the chemoradiotherapy group and from 30.0±1.0 to 24.4±1.3 in the surgery plus adjuvant RT group (both p<0.001). The mean change did not differ significantly between groups (5.4±1.3 vs. 5.6±1.0; p=0.471). Pain, dysphagia, bleeding or ulceration, and respiratory distress decreased during short-term follow-up in both arms.

Conclusions

Both treatment pathways were associated with short-term decreases in FACT-HNSI-10-derived raw symptom burden scores and improvements in symptom burden. No statistically significant difference was observed between groups in short-term functional improvement. These findings should be interpreted cautiously because of the small sample size, non-randomized design, and limited follow-up. They do not establish oncologic equivalence.

## Introduction

Oral cavity squamous cell carcinoma (OCSCC) is a major cause of cancer-related morbidity, particularly in South Asia, where tobacco use, betel nut chewing, smokeless tobacco use, and poor oral hygiene remain common risk factors [1,2]. Many patients present with locally advanced disease and experience symptoms that directly affect daily life, including pain, dysphagia, bleeding, ulceration, respiratory discomfort, impaired oral intake, and reduced functional well-being [3]. In Bangladesh, these difficulties are often compounded by late presentation, high patient volume, limited access to specialized surgical care, and financial constraints [4].

For resectable locally advanced OCSCC, surgery followed by adjuvant radiotherapy (RT), with or without concurrent chemotherapy depending on pathological risk factors, remains the conventional standard treatment [5,6]. This approach allows tumor removal and pathological risk stratification. However, extensive oral cavity surgery may require complex reconstruction and can be associated with postoperative morbidity, prolonged treatment time, impaired speech and swallowing, and delayed return to daily functioning [7]. In resource-limited settings, long surgical waiting times, limited operating facilities, reconstructive complexity, and the need for timely adjuvant therapy may further affect treatment delivery.

Definitive concurrent chemoradiotherapy (CCRT) is not generally considered a replacement for surgery in operable oral cavity cancer. However, it is used in selected patients who are medically unfit for surgery, unwilling to undergo surgery, or unable to undergo surgery within an appropriate timeframe [7,8]. Previous comparative studies have reported variable outcomes for definitive CCRT versus surgery followed by adjuvant RT in advanced oral cavity cancer. Some studies have favored primary surgery for disease control and survival, whereas others have suggested that definitive CCRT may provide acceptable outcomes in carefully selected patients [8-10]. These differences likely reflect variations in patient selection, tumor burden, treatment technique, treatment intent, and follow-up duration.

Although survival and locoregional control are central endpoints in oral cavity cancer research, they do not fully capture the early treatment experience of patients with symptomatic locally advanced disease. In resource-limited settings, where treatment selection may be shaped by surgical access, patient preference, and treatment burden, short-term symptom and functional outcomes are clinically important but less often reported [11]. This study, therefore, focused on patient-centered short-term outcomes using the Functional Assessment of Cancer Therapy Head and Neck Symptom Index-10-item version (FACT-HNSI-10), rather than attempting to establish oncologic equivalence [12,13].

This study aimed to compare short-term symptom and functional outcomes between patients with locally advanced OCSCC treated with definitive CCRT and those treated with surgery followed by adjuvant RT. The primary outcome was the change in the FACT-HNSI-10-derived raw symptom burden score from baseline to post-treatment assessment. Secondary outcomes included changes in pain, dysphagia, bleeding or ulceration, and respiratory distress. The study was designed as an exploratory comparison of short-term patient-centered outcomes in a small, non-randomized cohort and was not intended to establish treatment superiority, equivalence, or causal treatment effects between the two approaches.

This article was previously presented in part as an e-poster at the European Society for Medical Oncology (ESMO) Asia Congress 2025, held in Singapore from December 5 to 7, 2025.

## Materials and methods

Study design and setting

This prospective, non-randomized comparative cohort study evaluated two curative-intent treatment pathways in patients with locally advanced stage III or IVA OCSCC. The study was conducted in the Departments of Radiation Oncology at the National Institute of Cancer Research and Hospital (NICRH) and Ahsania Mission Cancer and General Hospital (AMCGH), Bangladesh, from January 2023 to October 2024. All patients were previously untreated and were followed according to the same institutional schedule for up to 24 weeks after treatment completion.

Study population and eligibility criteria

Sixty-eight patients were included in the final analysis: 34 received definitive CCRT (Arm A), and 34 underwent surgery followed by adjuvant RT (Arm B). Patients were allocated to treatment pathways based on clinical suitability, multidisciplinary decision-making, patient preference, and routine treatment planning rather than by random allocation; therefore, selection bias was possible.

Eligible patients had histologically confirmed OCSCC with clinical and/or radiological evidence of stage III or IVA disease. Exclusion criteria were an Eastern Cooperative Oncology Group (ECOG) performance status ≥3, synchronous malignancy, age <18 or >70 years, previous chemotherapy or RT, recurrent disease, pregnancy, major organ dysfunction, comorbidities precluding curative-intent treatment, or unwillingness to participate. Patients were discontinued from the study if they withdrew consent or developed unacceptable toxicity.

Patient assessment and data collection

Baseline assessment included demographic characteristics, risk factors, comorbidities, ECOG performance status, presenting symptoms, tumor site, T stage, N stage, and tumor-node-metastasis (TNM) stage. Pretreatment evaluation included history, clinical examination, histopathological confirmation, laboratory investigations, and imaging as indicated. Contrast-enhanced computed tomography (CT) of the face and neck was used for disease assessment, and staging followed the American Joint Committee on Cancer (AJCC) eighth edition system.

Data were collected using a pretested semi-structured questionnaire and recorded in a structured data form. The questionnaire was pretested among six patients in the NICRH outpatient department and revised before use. Symptom burden and functional status were assessed at baseline and at 24 weeks after treatment using the FACT-HNSI-10. For this analysis, responses were summarized as a FACT-HNSI-10-derived raw symptom burden score, in which higher scores indicated greater symptom burden and lower scores indicated lower symptom burden. The same scoring approach was applied consistently to all patients.

Before summation, positively worded functional items were reverse-coded so that all items were aligned in the same direction, with higher values indicating greater symptom burden. The final FACT-HNSI-10-derived raw symptom burden score included only the 10 FACT-HNSI-10 items and ranged from 0 to 40. The additional treatment side-effect item shown in Appendix was collected separately and was not included in the primary score.

Treatment pathways

Treatment decisions were made through a multidisciplinary approach involving radiation oncologists, maxillofacial surgical oncologists, and medical oncologists. Patients in Arm A received definitive CCRT. RT was delivered using a two-dimensional technique with a linear accelerator and a 6-MV photon beam. Patients were treated with parallel-opposed lateral fields after immobilization with a head and neck rest, shoulder retractor, and thermoplastic mask. The prescribed dose was 66 Gy in 33 fractions, delivered five days per week over approximately 45 days. Weekly cisplatin (40 mg/m²) was given concurrently when hematologic, renal, and general clinical parameters were acceptable.

Patients in Arm B underwent surgery followed by adjuvant RT. Surgical procedures included partial or hemiglossectomy, wide local excision, mandibulectomy, maxillectomy, and ipsilateral or bilateral neck dissection, depending on disease extent and surgical assessment. Adjuvant RT began approximately four to six weeks after surgery and was delivered to a dose of 60 Gy in 30 fractions, five days per week over approximately 42 days.

Supportive care was provided as required and included analgesics, antibiotics, antifungal agents, anti-ulcer medications, oral care, nutritional support, nasogastric feeding when indicated, dental evaluation before RT, and blood or blood component transfusion. Treatment interruptions, delayed RT completion, missed chemotherapy cycles, and major toxicities requiring treatment modification were recorded when present.

Outcome measures and follow-up

The primary outcome was the change in the FACT-HNSI-10-derived raw symptom burden score from baseline to the 24-week post-treatment assessment. Secondary outcomes included changes in pain, dysphagia, bleeding or ulceration, and respiratory distress. Symptoms were graded as absent, mild, moderate, or severe using predefined clinical criteria based on patient report and examination. Acute treatment-related toxicities, including skin reactions, mucositis, xerostomia, and anemia, were assessed using Radiation Therapy Oncology Group (RTOG) toxicity criteria.

Patients were assessed weekly during RT for symptom response and acute toxicity. Post-treatment follow-up was performed at six, 12, and 24 weeks. Patients who missed scheduled visits were contacted when possible, and available clinical records were reviewed. Missing follow-up data, if any, were documented and excluded from the relevant analyses. The assessment of locoregional recurrence was not included as a comparative endpoint because of the short follow-up duration and variable imaging availability.

Statistical analysis

Data were checked, coded, entered into a master sheet, and analyzed using IBM SPSS Statistics for Windows, version 27.0 (IBM Corp., Armonk, NY, USA). Continuous variables were expressed as mean ± standard deviation, and categorical variables as frequencies and percentages. An independent-samples t-test was used for between-group comparisons of continuous variables, and a paired-samples t-test was used for within-group pre- and post-treatment FACT-HNSI-10-derived raw symptom burden score comparisons. Categorical variables were analyzed using the chi-square test or Fisher’s exact test, as appropriate. Analyses were performed using available-case data, and no imputation was performed because of the small sample size and short follow-up period. A two-sided p-value of ≤0.05 was considered statistically significant.

Ethical considerations

The study was approved by the Institutional Review Boards of the NICRH and AMCGH, Dhaka, Bangladesh (approval nos. 2023/93 and 2023/55; approval date: January 26, 2023). Written informed consent was obtained from all participants before enrollment. The study was conducted in accordance with the ethical principles of the Declaration of Helsinki. Privacy and confidentiality were maintained throughout the study, and participants were informed about the study's purpose, procedures, potential risks and benefits, and their right to withdraw at any stage without affecting their treatment.

## Results

Patient characteristics

A total of 68 patients were included in the final analysis, with 34 patients in each treatment arm. Arm A received definitive CCRT, and Arm B underwent surgery followed by adjuvant RT. Baseline demographic and clinical characteristics are summarized in Table 1.

**Table 1:** Baseline demographic and clinical characteristics by treatment arm Data are presented as n (%) unless otherwise stated. P-values were calculated using the independent-samples t-test, chi-square test, or Fisher's exact test, as appropriate. CCRT: concurrent chemoradiotherapy; RT: radiotherapy; SD: standard deviation; TNM: tumor-node-metastasis

Characteristic	Arm A: definitive CCRT (n=34)	Arm B: surgery followed by adjuvant RT (n=34)	P-value
Age, years, mean±SD	54.7±9.3	58.6±8.5	0.073
Male sex	24 (70.6)	25 (73.5)	1
Smoking	14 (41.2)	11 (32.4)	0.615
Betel nut chewing	26 (76.5)	31 (91.2)	0.186
Chewing tobacco (jorda)	23 (67.6)	26 (76.5)	0.59
Primary tumor site			0.744
Tongue	11 (32.4)	11 (32.4)	
Buccal mucosa	11 (32.4)	9 (26.5)	
Upper gingiva	4 (11.8)	2 (5.9)	
Lower gingiva	5 (14.7)	6 (17.6)	
Retromolar trigone	3 (8.8)	6 (17.6)	
T stage			0.405
T2	11 (32.4)	8 (23.5)	
T3	15 (44.1)	13 (38.2)	
T4a	8 (23.5)	13 (38.2)	
N stage			0.144
N1	15 (44.1)	22 (64.7)	
N2	19 (55.9)	12 (35.3)	
TNM stage			1
Stage III	15 (44.1)	16 (47.1)	
Stage IVA	19 (55.9)	18 (52.9)	

The mean age was 54.7±9.3 years in Arm A and 58.6±8.5 years in Arm B (p=0.073). Most patients were male in both groups: 24 patients (70.6%) in Arm A and 25 patients (73.5%) in Arm B. Common risk factors included betel nut chewing, chewing tobacco, and smoking, with no statistically significant differences between the groups.

The tongue and buccal mucosa were the most frequent primary tumor sites. In Arm A, tongue and buccal mucosa lesions were each observed in 11 patients (32.4%). In Arm B, tongue lesions were seen in 11 patients (32.4%), followed by buccal mucosa lesions in nine patients (26.5%). Stage IVA disease was present in 19 patients (55.9%) in Arm A and 18 patients (52.9%) in Arm B. N2 disease was more frequent in Arm A than in Arm B, occurring in 19 patients (55.9%) and 12 patients (35.3%), respectively, although this difference was not statistically significant (p=0.144).

Baseline symptom burden

Baseline symptom burden is shown in Table 2. Pain was the most common presenting symptom, reported by 30 patients (88.2%) in Arm A and 27 patients (79.4%) in Arm B (p=0.512). Dysphagia was present in 18 patients (52.9%) in Arm A and 20 patients (58.8%) in Arm B (p=0.807). Respiratory distress was reported by six patients (17.6%) in each group. Bleeding or ulceration was uncommon, occurring in two patients (5.9%) in Arm A and one patient (2.9%) in Arm B. No statistically significant baseline differences in symptom presence were detected between treatment arms.

**Table 2:** Baseline symptom burden by treatment arm Indented rows show the baseline severity distribution. P-values are shown for symptom presence between treatment arms. CCRT: concurrent chemoradiotherapy; RT: radiotherapy

Baseline symptom	Arm A: definitive CCRT (n=34)	Arm B: surgery followed by adjuvant RT (n=34)	P-value
Pain present	30 (88.2)	27 (79.4)	0.512
None	4 (11.8)	7 (20.6)	
Mild	10 (29.4)	8 (23.5)	
Moderate	10 (29.4)	10 (29.4)	
Severe	10 (29.4)	9 (26.5)	
Dysphagia present	18 (52.9)	20 (58.8)	0.807
None	16 (47.1)	14 (41.2)	
Mild	10 (29.4)	9 (26.5)	
Moderate	6 (17.6)	9 (26.5)	
Severe	2 (5.9)	2 (5.9)	
Bleeding or ulceration present	2 (5.9)	1 (2.9)	1
None	32 (94.1)	33 (97.1)	
Mild	2 (5.9)	1 (2.9)	
Respiratory distress present	6 (17.6)	6 (17.6)	1
None	28 (82.4)	28 (82.4)	
Mild	4 (11.8)	4 (11.8)	
Moderate	2 (5.9)	2 (5.9)	

Functional Assessment of Cancer Therapy Head and Neck Symptom Index-10-item version outcomes

**Figure 1:**
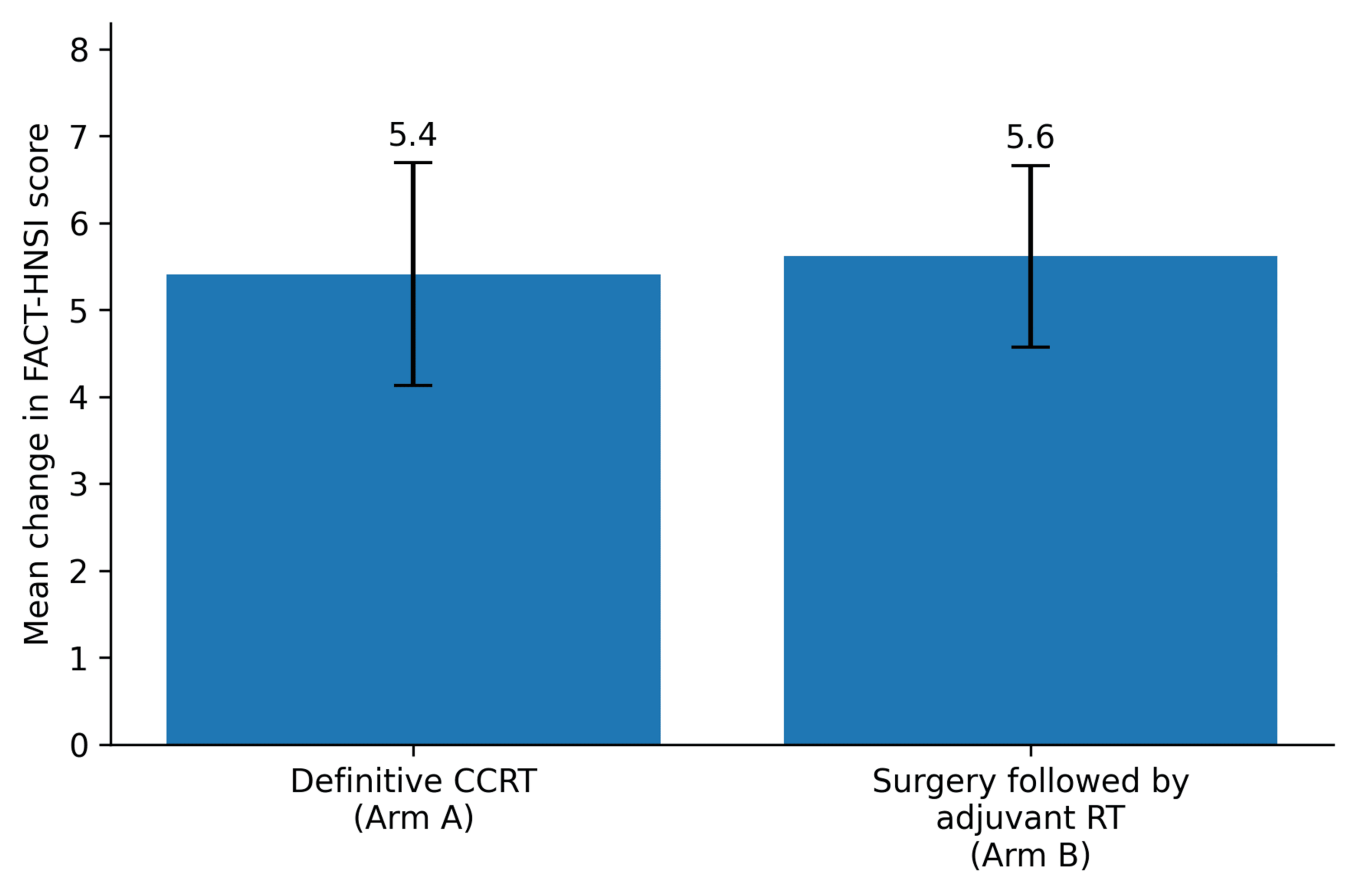
Mean decrease in the FACT-HNSI-10-derived raw symptom burden score by treatment arm Bars show the mean change from baseline to post-treatment assessment; error bars represent the standard deviation. FACT-HNSI-10: Functional Assessment of Cancer Therapy Head and Neck Symptom Index-10-item version; CCRT: concurrent chemoradiotherapy; RT: radiotherapy

FACT-HNSI-10-derived raw symptom burden score changes are summarized in Table 3 and Figure 1. These scores decreased significantly after treatment in both groups, indicating improvement in symptom burden according to the scoring approach used in this study. In Arm A, the mean score decreased from 30.2±0.8 before treatment to 24.8±1.3 after treatment (p<0.001). In Arm B, the mean score decreased from 30.0±1.0 to 24.4±1.3 (p<0.001). The mean change in the FACT-HNSI-10-derived raw symptom burden score was 5.4±1.3 in Arm A and 5.6±1.0 in Arm B, with no statistically significant between-group difference (p=0.471). Baseline and post-treatment FACT-HNSI-10-derived raw symptom burden scores were also comparable between groups (p=0.428 and p=0.222, respectively).

**Table 3:** Pre- and post-treatment FACT-HNSI-10-derived raw symptom burden scores Values are presented as mean±SD. Lower post-treatment scores reflected reduced symptom burden according to the scoring approach used in this study. Between-group p-values were calculated using the independent-samples t-test; within-arm p-values were calculated using the paired-samples t-test. FACT-HNSI-10: Functional Assessment of Cancer Therapy Head and Neck Symptom Index-10-item version; SD: standard deviation; CCRT: concurrent chemoradiotherapy; RT: radiotherapy

Measure	Arm A: definitive CCRT (n=34)	Arm B: surgery followed by adjuvant RT (n=34)	Between-group p-value
Baseline raw symptom burden score	30.2±0.8	30.0±1.0	0.428
Post-treatment raw symptom burden score	24.8±1.3	24.4±1.3	0.222
Mean change from baseline	5.4±1.3	5.6±1.0	0.471
Within-arm pre-post p-value	<0.001	<0.001	

Symptom response during follow-up

Symptom status at baseline and 24-week follow-up is presented in Table 4. Pain decreased in both arms during follow-up. At baseline, severe pain was present in 10 patients (29.4%) in Arm A and nine patients (26.5%) in Arm B. At 24 weeks, no patient in either arm had moderate or severe pain. Pain was absent in 25 patients (73.5%) in Arm A and 31 patients (91.2%) in Arm B; the remaining patients reported only mild pain.

**Table 4:** Symptom status at baseline and 24-week follow-up Data are presented as n (%) using the full treatment-arm denominator. The 24-week p-value compares residual symptom presence between treatment arms using Fisher’s exact test.

Symptom	Arm	Baseline present	Symptom-free at 24 weeks	Residual mild symptom at 24 weeks	Moderate/severe at 24 weeks	24-week p-value
Pain	Arm A	30 (88.2)	25 (73.5)	9 (26.5)	0 (0.0)	0.109
	Arm B	27 (79.4)	31 (91.2)	3 (8.8)	0 (0.0)	
Dysphagia	Arm A	18 (52.9)	31 (91.2)	3 (8.8)	0 (0.0)	0.239
	Arm B	20 (58.8)	34 (100.0)	0 (0.0)	0 (0.0)	
Bleeding or ulceration	Arm A	2 (5.9)	34 (100.0)	0 (0.0)	0 (0.0)	1
	Arm B	1 (2.9)	34 (100.0)	0 (0.0)	0 (0.0)	
Respiratory distress	Arm A	6 (17.6)	34 (100.0)	0 (0.0)	0 (0.0)	1
	Arm B	6 (17.6)	34 (100.0)	0 (0.0)	0 (0.0)	

Dysphagia also decreased over time. At baseline, dysphagia was present in 18 patients (52.9%) in Arm A and 20 patients (58.8%) in Arm B. At 24 weeks, only three patients (8.8%) in Arm A had mild dysphagia, while all patients in Arm B were free of dysphagia. No patient had moderate or severe dysphagia at the final follow-up.

Bleeding or ulceration resolved in both groups. Mild bleeding or ulceration was present at baseline in two patients (5.9%) in Arm A and one patient (2.9%) in Arm B. No bleeding or ulceration was observed in either group at three or six months. Respiratory distress also resolved after treatment; by three and six months, no patient in either arm had respiratory distress.

Treatment-related toxicities

Acute toxicities were observed in both groups and generally improved during follow-up. During the later phase of RT, skin toxicity was present in 13 patients (38.2%) in Arm A and 15 patients (44.1%) in Arm B. Mucositis was the most common toxicity, affecting 22 patients (64.7%) in Arm A and 21 patients (61.8%) in Arm B. By six months, skin toxicity and mucositis had resolved in all patients. Xerostomia persisted longer than skin toxicity and mucositis. At three months, xerostomia was present in 10 patients (29.4%) in Arm A and 11 patients (32.4%) in Arm B. At six months, it persisted in five patients (14.7%) and six patients (17.6%), respectively. Anemia was uncommon and was reported in three patients (8.8%) in Arm A and seven patients (20.6%) in Arm B during the early follow-up period.

## Discussion

This study compared short-term symptom and functional outcomes in patients with locally advanced OCSCC treated with definitive CCRT or surgery followed by adjuvant RT. FACT-HNSI-10-derived raw symptom burden scores decreased significantly after treatment in both groups, indicating improvement in symptom burden, with no statistically significant difference in the magnitude of improvement between treatment arms. Pain, dysphagia, bleeding or ulceration, and respiratory distress also decreased during follow-up. These findings suggest that both treatment pathways were associated with short-term symptomatic and functional improvement in this cohort. However, because this was a small, non-randomized study with limited follow-up, the results should not be interpreted as evidence that definitive CCRT is oncologically equivalent to surgery-based treatment.

Surgery followed by adjuvant RT, with or without chemotherapy depending on pathological risk factors, remains the standard approach for operable, locally advanced oral cavity cancer [5,14]. Surgery offers tumor removal, pathological staging, and risk-adapted postoperative treatment. At the same time, extensive oral cavity surgery may require complex reconstruction and can affect swallowing, speech, oral intake, body image, and return to daily functioning [8,15,16]. These concerns are especially relevant in resource-limited settings, where surgical waiting times, cost, access to reconstructive expertise, and timely delivery of adjuvant therapy may influence treatment selection.

Definitive CCRT is not generally considered a substitute for surgery in operable oral cavity cancer. Still, it remains an important option for selected patients who are medically unfit for surgery, unwilling to undergo surgery, or unable to receive timely surgical care [7,8,17]. Previous comparative studies have reported mixed outcomes. Some have favored surgery for survival and disease control, while others have suggested that definitive CCRT can provide acceptable outcomes in carefully selected patients [8-10,16]. Differences across studies likely reflect variation in tumor burden, operability, patient selection, radiation technique, chemotherapy use, and follow-up duration. The present study, therefore, contributes most appropriately to short-term, patient-centered outcome data rather than to definitive survival comparisons.

The decrease in FACT-HNSI-10-derived raw symptom burden scores in both arms suggests improvement in reported symptoms during follow-up. The FACT-HNSI-10-derived raw symptom burden scores are designed to assess priority symptoms and functional concerns in head and neck cancer, including pain, swallowing, eating, breathing, communication, and quality-related concerns [12,18]. In this study, FACT-HNSI-10-derived raw symptom burden scores decreased after treatment, as measured by the dataset's scoring approach, indicating reduced symptom burden. The absence of a significant between-group difference suggests that, over short-term follow-up, both treatment pathways were associated with comparable functional improvement as measured by this instrument. This matters because patients with locally advanced oral cavity cancer often present with symptoms that directly affect nutrition, comfort, and treatment tolerance [7,13].

Pain was the most frequent presenting symptom and decreased in both groups. By 24 weeks, no patient had moderate or severe pain, and most patients were pain-free. Dysphagia also decreased, with only three patients in the CCRT arm reporting mild dysphagia at final follow-up. Bleeding or ulceration and respiratory distress resolved in both groups. These findings support the value of reporting symptom-based outcomes separately from survival endpoints, particularly in patients whose treatment decisions are shaped by fitness for surgery, access to care, and personal preference [7,8,18].

Treatment-related toxicities were common but generally manageable. Mucositis was the most frequent acute toxicity, followed by skin reaction and xerostomia. Skin toxicity and mucositis resolved by six months, while xerostomia persisted in a smaller proportion of patients. This pattern is consistent with the known toxicity profile of head and neck RT, especially when delivered with concurrent chemotherapy or after surgery [5,16]. The use of two-dimensional RT should be considered when interpreting these findings, as modern conformal techniques may better spare normal tissues and reduce late toxicity [14].

Several limitations should be acknowledged. First, the sample size was small, which limits statistical power and increases the risk of type II error. Second, treatment allocation was non-randomized and based on clinical suitability, surgical fitness, comorbidity, and patient preference; therefore, selection bias and residual confounding are unavoidable. Because treatment allocation was based on clinical suitability, surgical fitness, comorbidity, and patient preference, confounding by indication cannot be excluded. Third, follow-up was limited to 24 weeks, which is not sufficient to assess long-term survival, durable locoregional control, late toxicity, or long-term quality of life. Fourth, the study was conducted in two centers in Bangladesh using a two-dimensional RT technique, which may limit generalizability to centers using modern intensity-modulated RT. Finally, although FACT-HNSI-10-derived raw symptom burden scores provided useful symptom-focused information, a broader quality-of-life instrument and longer follow-up would provide a more complete assessment of recovery. Because symptom assessment was not blinded, responder bias cannot be excluded. In addition, inclusion of two centers may have introduced institutional variability in surgical practice, RT delivery, and supportive care.

Despite these limitations, the study addresses a clinically relevant question in a resource-limited setting. Both definitive CCRT and surgery followed by adjuvant RT were associated with significant short-term decreases in FACT-HNSI-10-derived raw symptom burden scores. No statistically significant between-group difference was observed in the change in the FACT-HNSI-10-derived raw symptom burden score during short-term follow-up. Given the small sample size and non-randomized design, this finding should not be interpreted as evidence of equivalence between treatment approaches. These findings should be interpreted cautiously and should not be used to claim equivalence between definitive CCRT and surgery-based standard treatment. Larger prospective studies with balanced treatment groups, modern RT techniques, standardized patient-reported outcomes, and longer follow-up are needed.

## Conclusions

In this small non-randomized cohort, FACT-HNSI-10-derived raw symptom burden scores decreased after treatment in both patients receiving definitive CCRT and those undergoing surgery followed by adjuvant RT, indicating improvement in symptom burden. Pain, dysphagia, bleeding or ulceration, and respiratory distress decreased during short-term follow-up in both groups.

No statistically significant difference was observed between the two treatment arms in short-term functional improvement. These findings should be interpreted cautiously because of the non-randomized design, small sample size, and limited follow-up. The results do not establish oncologic equivalence, and larger prospective studies with longer follow-up are needed.

## Figures and Tables

**Figure 2:**
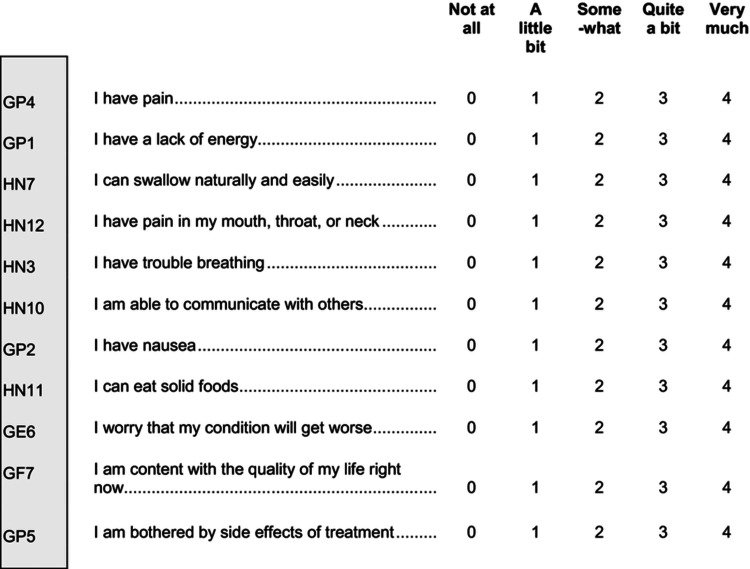
FACT-HNSI-10 items and additional treatment side-effect item The figure shows the FACT-HNSI-10 response format. Items are rated on a five-point Likert scale from 0 (not at all) to 4 (very much). The displayed items assess pain, energy, swallowing, oral or throat discomfort, breathing, communication, nausea, the ability to eat solid foods, worry about disease progression, quality of life, and treatment-related side effects. FACT-HNSI-10: Functional Assessment of Cancer Therapy Head and Neck Symptom Index-10-item version; GP: general physical; GE: general emotional; GF: general functional; HN: head neck
